# LAGOS: learning health systems and how they can integrate with patient care

**DOI:** 10.1136/bmjhci-2019-100037

**Published:** 2019-10-15

**Authors:** Scott McLachlan, Kudakwashe Dube, Evangelia Kyrimi, Norman Fenton

**Affiliations:** 1 EECS, Queen Mary University of London, London, UK; 2 School of Fundamental Sciences, Massey University, Palmerston North, New Zealand

**Keywords:** BMJ Health Informatics, information science, information systems, public health, medical informatics

## Abstract

**Problem:**

Learning health systems (LHS) are an underexplored concept. How LHS will operate in clinical practice is not well understood. This paper investigates the relationships between LHS, clinical care process specifications (CCPS) and the established levels of medical practice to enable LHS integration into daily healthcare practice.

**Methods:**

Concept analysis and thematic analysis were used to develop an LHS characterisation. Pathway theory was used to create a framework by relating LHS, CCPS, health information systems and the levels of medical practice. A case study approach evaluates the framework in an established health informatics project.

**Results:**

Five concepts were identified and used to define the LHS learning cycle. A framework was developed with five pathways, each having three levels of practice specificity spanning population to precision medicine. The framework was evaluated through application to case studies not previously understood to be LHS.

**Discussion:**

Clinicians show limited understanding of LHS, increasing resistance and limiting adoption and integration into care routine. Evaluation of the presented framework demonstrates that its use enables: (1) correct analysis and characterisation of LHS; (2) alignment and integration into the healthcare conceptual setting; (3) identification of the degree and level of patient application; and (4) impact on the overall healthcare system.

**Conclusion:**

This paper contributes a theoretical framework for analysis, characterisation and use of LHS. The framework allows clinicians and informaticians to correctly identify, characterise and integrate LHS within their daily routine. The overall contribution improves understanding, practice and evaluation of the LHS application in healthcare.

**Summary**

**What is already known?**

- Learning health systems (LHS) embody the relationship between care practice, research and knowledge translation.
- LHS are recognised to be one of the major computing technological advances in healthcare.
- LHS are a recent concept and are still not well understood among clinicians which limits successful implementation in practice.
- Even with taxonomic classification for LHS types, there is still a gap in that there is limited knowledge to allow LHS to be described in terms of where they operate or interact with activities of patient care.

**What does this paper add?**

- Five concepts are identified to define contemporary LHS.
- The LAGOS framework is developed with five pathways, each with three levels of practice specificity, to bring together research efforts in the domains of LHS, clinical care process specification and population and precision medicine.
- The taxonomy and LAGOS framework are evaluated through application to case studies not previously understood to be LHS.

## Introduction

Learning health systems (LHS) embody the relationship between care practice, research and knowledge translation^1^ and are recognised as one of the major computing technological advances in healthcare.^2–4^ However, most work published on LHS dates from 2011^5^ although many works which do not explicitly mention LHS also fall within the domain.^6^ Unsurprisingly, the LHS concept is still not well understood and there has been no coherent work to: (1) align LHS with clinical practice; and (2) identify how LHS will operate, interact and integrate with practical patient care. This prevents formation of the critical mass of research effort needed for LHS.^6^ This paper addresses the limited understanding and lack of conceptual and theoretical tools for LHS adoption and application in healthcare practice.

We have previously investigated the aspects of LHS^5–7^ and common *clinical care process specification* (CCPS) documents and their application to medical practice.^8 9^ However, to the best of our knowledge, this paper is the first to attempt to integrate these into a single unified framework that can improve understanding and success in health informatics and LHS implementation. This paper presents a new unified and holistic framework, LAGOS, for LHS and demonstrates application of the framework within the context of a case study (CS) from an Engineering and Physical Sciences Research Council funded project, PamBayesian.^10^

LHS are a significant evolution from evidence-based medicine (EBM). Greater awareness of LHS is necessary to achieve success in the goal of delivering precision medicine. Potentially, LHS may be used in a wide range of systems and application domains, providing benefits to all areas of healthcare. Use of this approach, taxonomy and framework helps address challenges in realising all LHS' potential.

## Methods and materials

The *literature collection* covering LHS,^5^ electronic health record (EHR)^7^ and CCPS documents^9^ were reviewed using *concept analysis* (CA) and *thematic analysis* (TA). *Pathway theory* with a *layer-based architectural perspective* 11 was used to develop the LAGOS framework. The resulting framework was evaluated using a *CS approach* looking at PamBayesian.^10^ Table 1 presents a mapping of the methodologies used in the conduct of this research.

**Table 1:** Summary of mapping between methodologies used and deliverables and objectives

Objective	Deliverable	Methodology
Objective 1	Approach to characterisation of learning health systems	Taxonomy developed from the literature using: concept analysis and thematic analysis. Abstraction of the clinical learning life-cycle into a design thinking-based perspective.
Objective 2	Framework	Pathway-based perspective applied together with taxonomy to well-known ‘*levels of medical practice*’
Objective 3	Evaluation	Three case studies that demonstrate the framework for learning health systems

### CA and TA

CA is a systematic coding and categorisation method for investigating texts and resolving quantitative description of features.^12 13^ TA provides the systematic element characteristic of CA, while also combining analysis of frequency with analysis of *in context* meaning, providing a more truly qualitative analysis.^12^ CA and TA are established methodologies regularly used in clinical, nursing and other healthcare research contexts.^12–15^ In this paper, CA of the definitions for LHS provided in the literature identified a large number of related concepts. TA of these concepts refined the key themes that define the LHS and identified their relationships within the context of LHS.

### The pathway and layer-based perspective

Development of a pathway theory and analytical framework can highlight underlying values and fundamental relationships between otherwise fragmented concepts.^16^ Different from the clinical or treatment pathways common to medicine and nursing, pathway theory is designed around consistent values and beliefs,^16^ in this case, the established *levels of medical practice application*.^11 17 18^ Starting with this *application pathway* and using it as the basis for ordering all other pathways, we sought to bring together the domains of *LHS* and *CCPS documentation* and arrange them on the basis of how each applies to the provision of *population*, *evidence-based* or *precision medicine*.

### Case studies

CS are a method for conducting and presenting comparative research into subject areas that include qualitative and mixed-mode information science inquiry.^19^ CS are frequently used within information science and are considered to be as well-developed as any other scientific method.^20^ A range of CS types exist, including: exploratory, explanatory, descriptive, intrinsic, instrumental and collective.^21 22^ CS allow researchers to capture the knowledge of practitioners using a broad variety of data sources to ensure the knowledge is considered through multiple lenses.^23 24^ CS in computing and information sciences tend to be more open methodologically, accepting of methods from both natural and social sciences. In this way they tend towards being more hybrid in nature when contrasted to those in the medical sciences that rely more on interviews and data collection techniques involving individuals. The taxonomy and LAGOS framework are evaluated in the context of three subprojects of the PamBayesian project.

## Results

### Characterisation and conceptualisation of LHS

Most LHS papers define LHS using definitions proposed in two seminal Institute of Medicine (IoM) workshop publications. Prior to the first contemporary LHS, the IoM described LHS as a system *in which knowledge generation is so embedded into the core of the practice of medicine that it is a natural outgrowth and product of the healthcare delivery process and leads to continual improvement in care*.^25^ Their latter and more highly cited definition describes an LHS as a system *in which progress in science, informatics and care culture align to generate new knowledge as an ongoing natural by-product of the care experience, and seamlessly refine and deliver best practices for continuous improvement in health and healthcare*.^26^ This definition fails to describe attributes that would contribute to aspects of patient care that LHS systems should target: *quality, safety, efficiency* and *efficacy*. As a consequence, the LHS domain has seen little development of these aspects.^5 6^ Similarly, the IoM definition does little to develop an understanding of the attributes and concepts that underpin implementation and usage of LHS in clinical practice.^5^

The unified conceptualisation and framework for systematically characterising LHS derives from our following strands of previous research:

- *Concept*: The lack of understanding of the *concept of LHS* was described as the *research community awareness challenge.* 6
- *Taxonomy*: Developed a taxonomy describing the entire scope of LHS as observed in the current literature showing how each type positions within the larger learning health organisation.^5^
- *Framework*: Developed a unifying framework showing the role and context for LHS and its integration into the learning healthcare organisation.^5^
- *Implementation*: Identified the benefits, barriers and potential facilitators for LHS, and comparative analysis of how these may have arisen from, or be related to, those that have impacted EHR implementation during the preceding 30 years.^7^
- *Realisation*: In this paper we demonstrate how the taxonomy, framework and factors for successful LHS implementation can be applied to a contemporary project to identify those aspects that constitute LHS, the type of LHS, the barriers to which facilitators may be applied and the benefits that may result.

**Figure 1:**
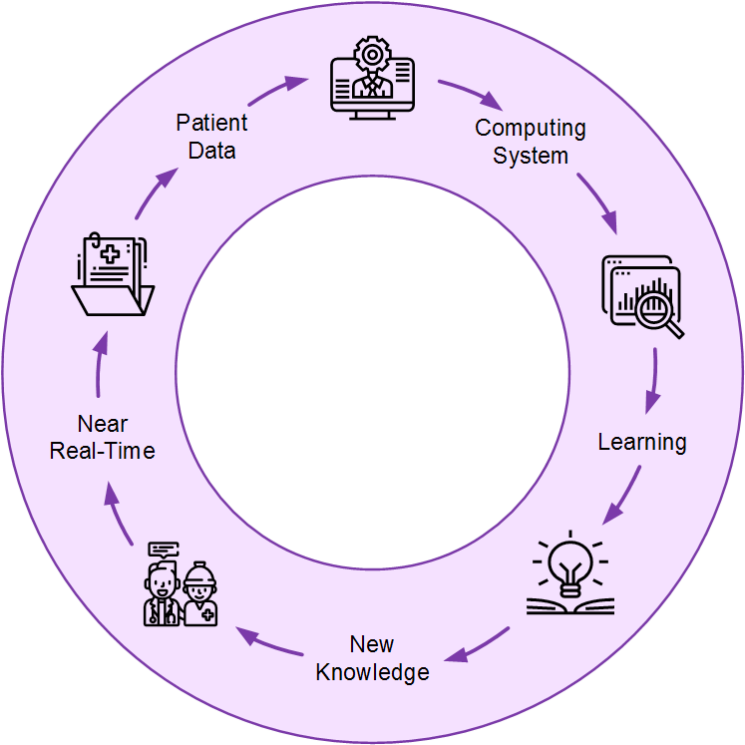
Five concepts that define learning health systems.

From the concepts identified in LHS definitions found across literature where authors self-identified their works as LHS, five themes converged which self-align into a learning life-cycle shown in figure 1. Similarly, EBM is best presented as a learning life-cycle which comprised five themes: assess, ask, acquire, appraise and apply.^27^ We propose that the themes defining LHS and *precision medicine* are also a form of a life-cycle consisting of the following five phases:

- *Patient data*: Collections of *patient data* in the form of EHR are seen as a near-inexhaustible source from which to learn new knowledge.^28 29^
- *Computing system*: Not limited to the computers used to access or store patient data, but also includes those which contain the programmes and perform the machine learning, prediction and other computing necessary to learn and apply knowledge.^29 30^
- *Learning*: The concept of *learning* as it relates to LHS are the processes that analyse data to derive or generate new knowledge.^28–31^
- *New knowledge*: The *new knowledge* learnt from patient data can advance our understanding of the underlying mechanisms of disease and patients’ response to treatment.^29 32^
- *Near real* *time*: The current driving ambition for LHS is to expedite the process, often described in terms of a 17-year lag, of getting knowledge to inform clinical decisions from scientific discovery to clinical use.^28 29 32 33^

### The LAGOS framework

**Figure 2:**
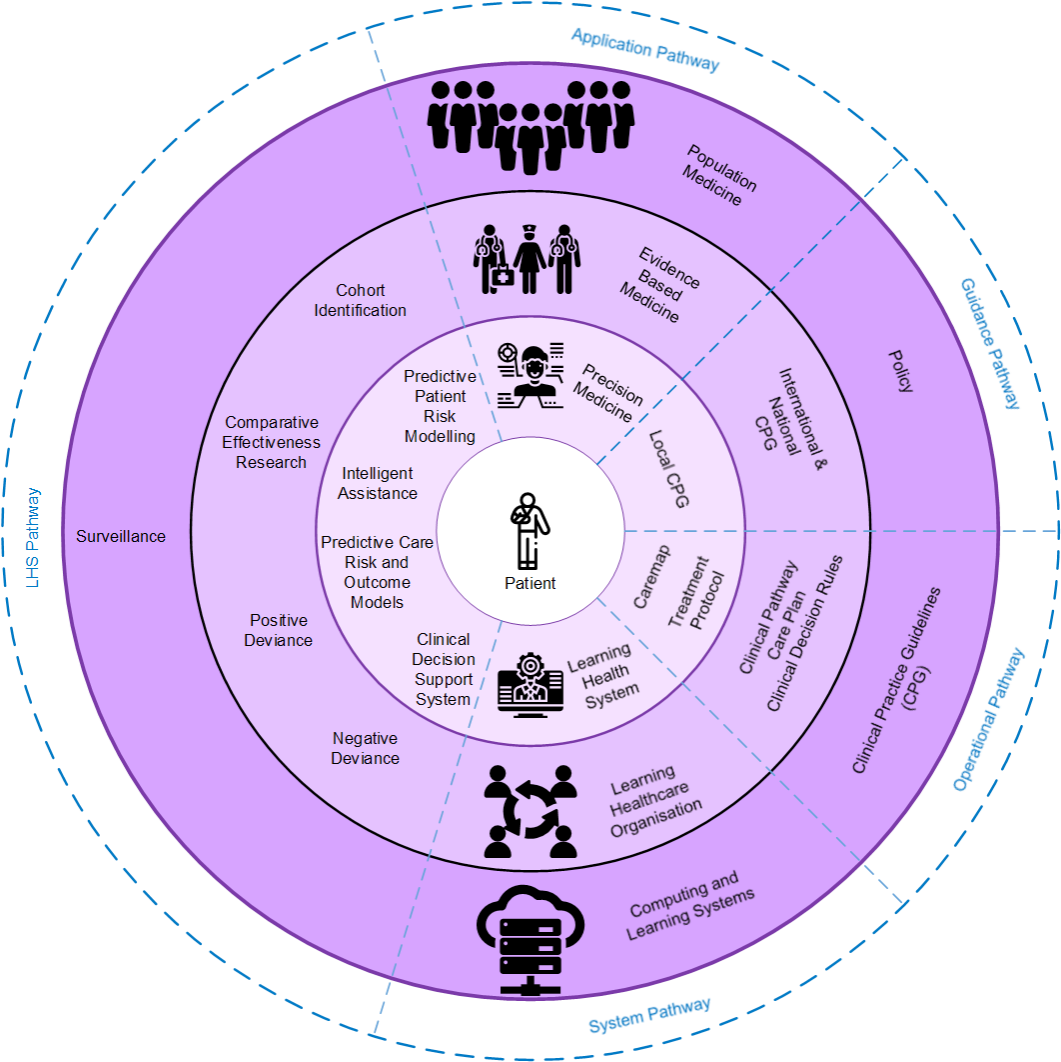
The LAGOS framework. LHS, learning health systems.

*LAGOS* is an acronym for the five pathways shown in figure 2. These pathways are *L*earning health systems, *A*pplications, *G*uidance, *O*perational and *S*ystems. They focus and converge on the patient, and broadly define the areas covered by the Framework. The intended focus of LAGOS is the individual patient presenting before the clinician. Each pathway radiates from the most general or broadest application of that pathway’s scope, towards the centre, which represents the most specific application, and which is directed towards the individual patient. Thus, within LAGOS, as the viewer moves along each pathway towards the patient in the centre, the focus of elements at each layer shifts from a population-based focus to a precision medicine focus. Precision medicine itself forms part of a life-cycle whenever the knowledge learnt from engaging LHS influences or impacts future decisions on health policy, population medicine, clinical practice guideline (CPG) or the development of new health-based computing and learning systems.

#### The application pathway

The pathway of medical practice and its ongoing shift from *population medicine* through EBM towards *precision medicine* is best described in the title of Horwitz *et al*’s^17^ paper, *From Evidence-Based Medicine to Medicine-Based Evidence*. Population medicine effectively promotes those activities that will improve general health for an entire population,^34^ is impacted and influenced by policy and financial concerns^34^ and is not always informed by clear or convincing scientific evidence.^35 36^ EBM focuses on informing clinicians with scientifically proven current best treatment options for a particular condition. This has sometimes been characterised as a *one-size-fits-all* approach.^11 18^ Precision medicine seeks to customise medical treatment by accounting for patient-specific factors in considering treatment options.^11^ Just as EBM is the scientific basis for, and epidemiological application of, population medicine,^17 37^ precision medicine is seen as the natural scientific evolution of EBM.^11^

#### The system pathway

The system pathway demonstrates the need for technology and learning in provision and improvement of healthcare. In the outer arc lie both the computing technologies and learning systems on which everything else, including EHR of the healthcare-consuming population, operate. On the next level, the learning organisation employs learning approaches and stored EHRs in the task of developing new evidence-based knowledge and treatments. Proximal to the patient on this pathway are the LHS; those systems presenting customised treatments for individual patients.

#### The LHS pathway

**Figure 3:**
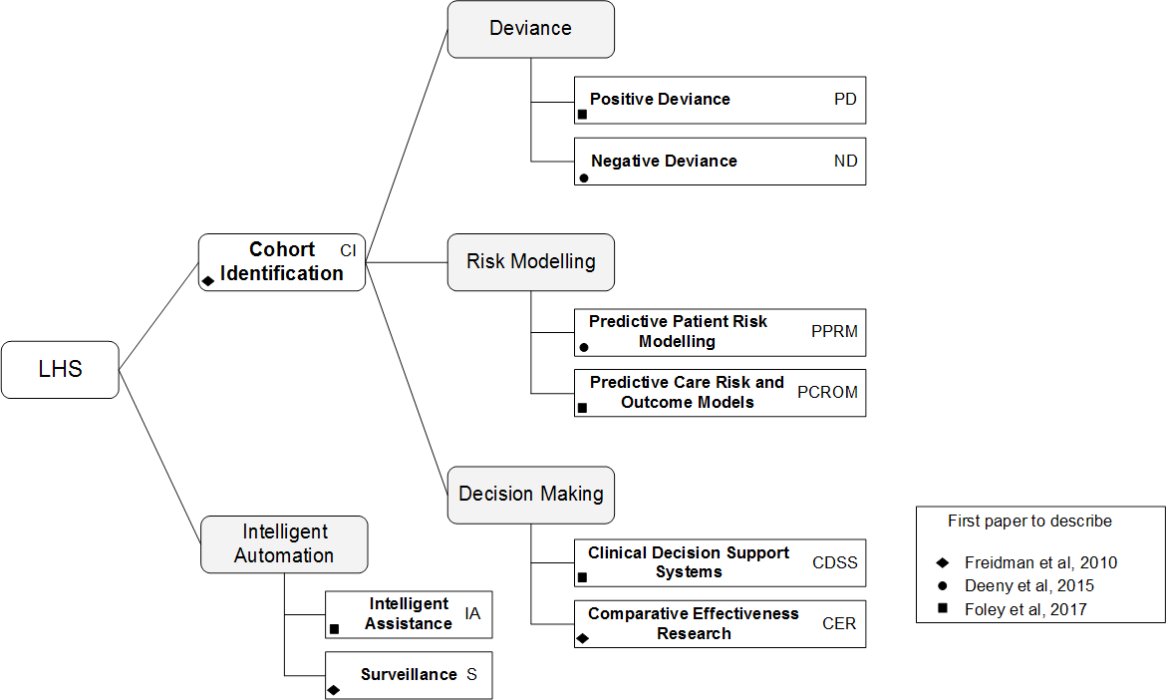
Learning health systems (LHS) taxonomy.^5^

The complete LHS taxonomy shown in figure 3 is represented in the LHS pathway of LAGOS. At the outer edge is *surveillance*, which operates as an automated alert process within information technology systems that (A) monitors the entire population’s EHR for diagnosis or clinical coding of a range of communicable diseases, and (B) can also be programmed to monitor for adverse treatment outcomes. Those LHS types at the second layer primarily work with or on the basis of EBM, or are used by clinicians in review of their, or other clinicians, treatment outcomes. This includes *cohort identification* which is most often used within learning health organisations to identify groups of patients based on one or more similar characteristics. Below are those LHS most proximal to the patient and which can be engaged by the clinician in direct patient care. These models support personalised clinical decision-making and predict risks and outcomes that may result for an individual patient from receiving the selected treatment. It is these LHS that directly meet the definition of being *precision medicine*.

#### The guidance and operational pathways

**Figure 4:**
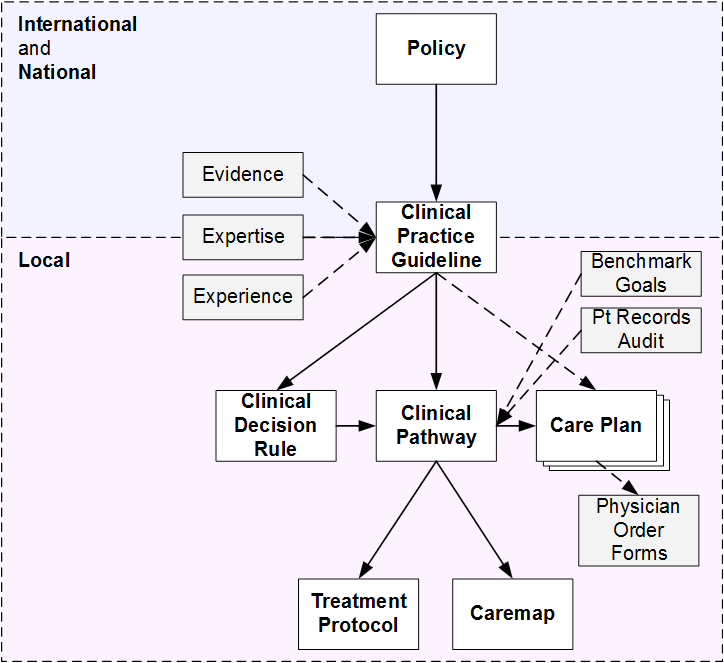
Taxonomy and hierarchy for clinical care process specifications.^9^

The full details of our process of developing the taxonomy, definitions and characterisations for CCPS are described in ref 9. Here we provide a brief summary necessary for the reader to understand the pathways. CCPS define healthcare policy and procedure and are arranged in a hierarchy describing both their operational nature and distance from the patient, as shown in figure 4. For example, *policy* is both the furthest from the individual patient in that it is set by governments to guide health services for entire populations, and least operational in that it is the most general document and least likely to be based on evidence-based science. CCPS can also be described based on whether their primary intention is guidance or operationalisation. Policy and the levels of CPG operate primarily in the guidance space and are described on the guidance pathway based on their proximity to the patient; policy is primarily population based while local CPGs are closest to the individual patient. There is overlap between the guidance and operational pathways in that local CPGs can also be seen with operational content. On the operational pathway, local CPGs lie at the population end as they would be applied to the general population of a health district diagnosed with a particular condition, while care plans, care maps and treatment protocols are found along the pathway converging towards individual patient treatment.

## Evaluation and discussion: *the PamBayesian project*

### LHS in the context of generating the Realistic Synthetic Electronic Health Record

Accessing EHR for secondary use purposes such as data research, modelling and artificial intelligence training presents with challenges, notably:

1. Attaining ethics approval for access to collections of EHR.
2. Difficulty when consent is required from each individual patient.
3. Over-reliance on anonymisation that can reduce or remove important contextual detail.

**Figure 5:**
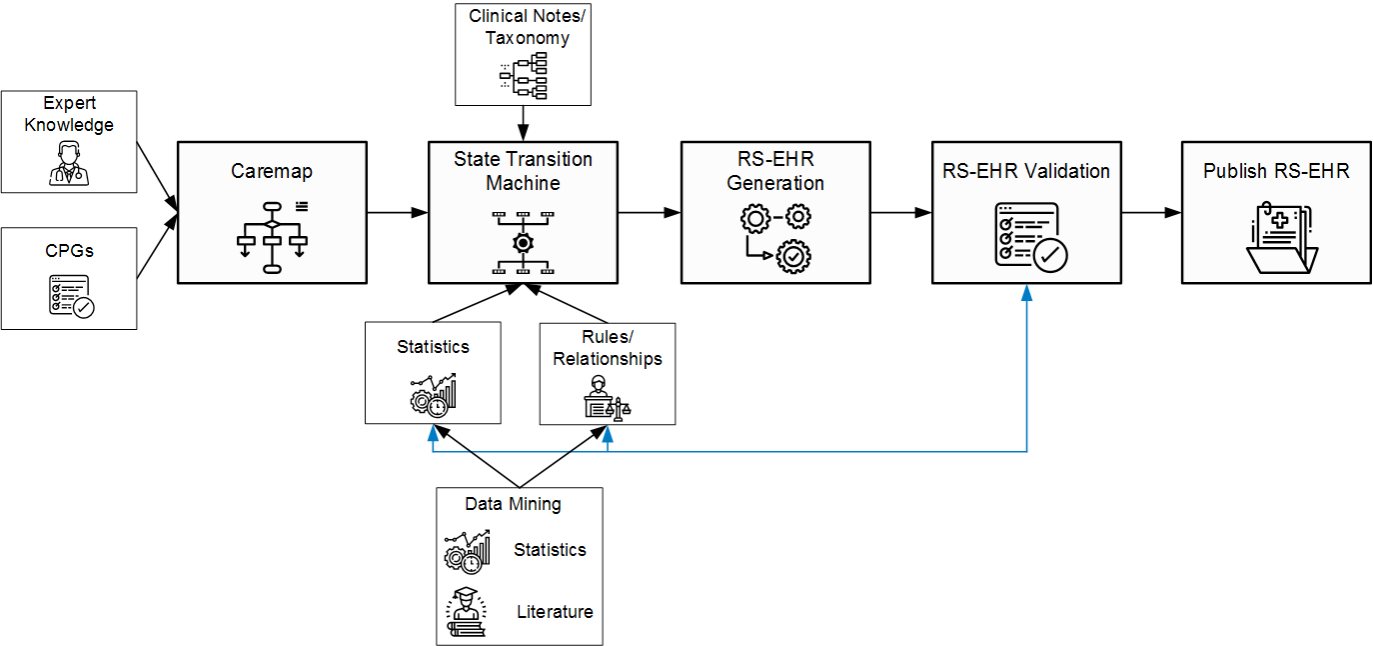
The ATEN approach to RS-EHR generation. CPG, clinical practice guideline; RS-EHR, Realistic Synthetic Electronic Health Record.

The *CoMSER Realistic Synthetic Electronic Health Record (RS-EHR*) and *ATEN Realism in Synthetic Data* projects operate following the approach described in figure 5 and focus on satisfying the need for access to EHR for secondary uses relying on a privacy-preserving knowledge-intensive method to generate locally realistic, but not real, synthetic EHR without needing access to the real EHR.

The relationship between LHS and RS-EHR can be two-way. LHS can help provide the aggregated statistical data and knowledge described as rules and relationships that exist in EHR data sets. In this way, RS-EHR generation need never be exposed to real EHR during the definition or generation of synthetic EHR. Conversely, LHS can be built, trained and validated by projects like PamBayesian using collections of RS-EHR, prior to being productionised to work for real patients and clinicians.

*The LHS paradigm allowed us to fully exploit the routinely collected data from the healthcare system*. This made development of knowledge-intensive methods for generating synthetic EHR successful, making it easy to create collections of realistic synthetic EHR for use in secondary uses where privacy concerns prevent release of real data. Further, development of knowledge-intensive models enables prediction of patient risk for particular negative outcomes and recommending appropriate and more effective treatments based on patient characteristics, history and current symptomatology possible.

To fulfil RS-EHR’s aims the following LHS types from the LHS pathway, which apply to the levels of *medical practice* from the application pathway (in brackets), are needed:

1. *Cohort identification—learning evidence* (EBM) and operating within the context of the *learning healthcare organisation* level of the *system pathway* to identify a prescribed cohort of patients with similar health conditions or characteristics such as demographics and symptomatology consistent with the disease to be modelled and generated.
2. *Positive deviance* and *negative deviance—learning evidence* (EBM) and operating within the context of the *learning healthcare organisation* level of the *system pathway*; of commonly used treatments, both effective and ineffective to ensure synthetic patients receive realistic treatments and outcomes.
3. *Predictive patient risk modelling—specific to patient* (precision medicine) and operating within the *LHS* level of the *system pathway* to identify patterns and model risk factors consistent with adverse events.
4. *Clinical decision support system—specific to patient* (precision medicine) and operating within the *LHS* level of the *system pathway* to identify characteristics of synthetic patients that make them compatible for generation of specific disease or treatment outcomes.

### LHS in the context of patient risk and decision modelling

There are numerous approaches for developing intelligent systems supporting clinical decision-making for diagnosis, prognosis or treatment selection. Bayesian networks (BNs) are one such approach. BNs model uncertainty and allow the user to update prior belief, such as when assessing the probability for presence of a medical condition in light of new evidence (additional symptoms, risk factors and test results). However, the process of building these intelligent systems for chronic conditions is not yet fully explored and understood. Chronic conditions are particularly challenging in this context as the patient’s condition must be monitored for extended periods during which many decisions may be undertaken. Ideally, doctors and nurses should be able to monitor patients without the resource-intensive, expense and inconvenience of clinic visits, except when such visits are necessary. Current clinical records and care processes do not easily receive, integrate or enable patients in the home to collect and transmit self-monitoring data from inexpensive sensor-based devices like the Apple Watch and continuous glucose monitors.

**Figure 6:**
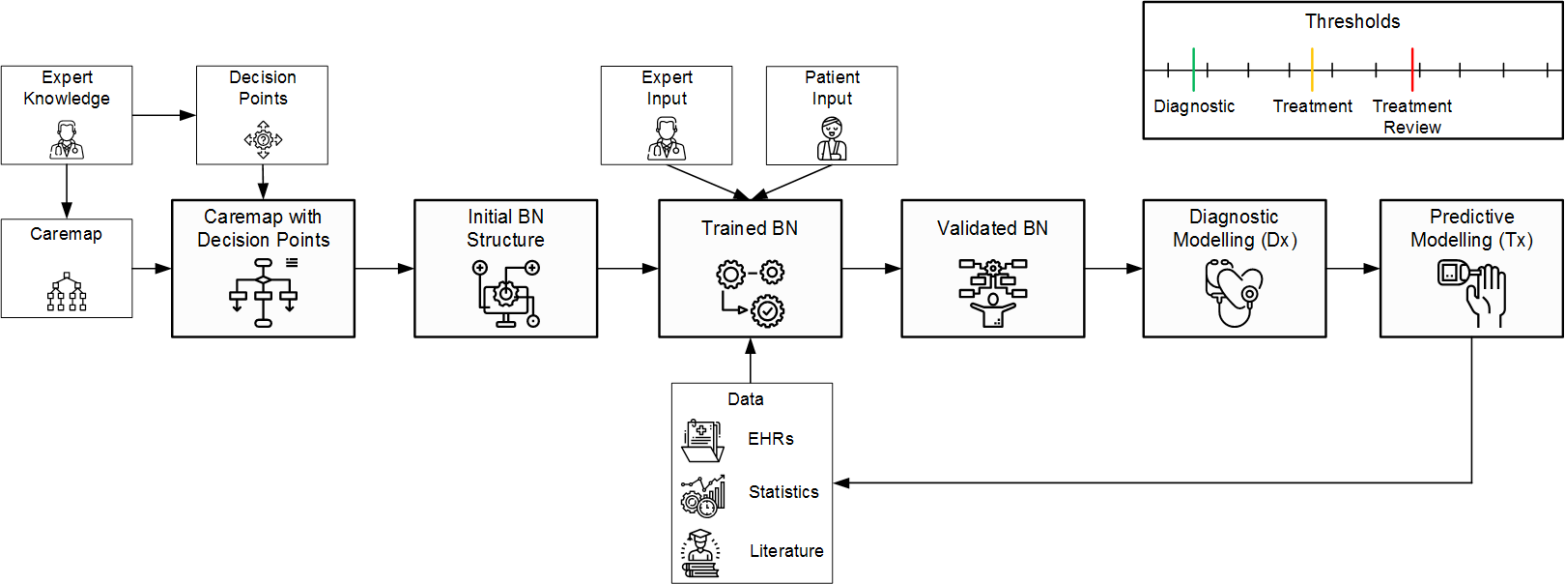
The PamBayesian project as a learning health system (LHS). BN, Bayesian network; EHR, electronic health record.

PamBayesian is developing a new framework for distributed probabilistic decision-support systems. As shown in figure 6, PamBayesian combines patient data with clinical expertise and patient input, for use in developing intelligent systems. The novelty of this framework is the use of ‘conventional’ EHR (eg, blood tests, imaging results) combined with near real-time continuous data from local sensors for learning and providing new knowledge. This allows for autonomy in a collaborative decision-making environment that includes clinicians and patients, to avoid unnecessary visits to a clinic or hospital. Once the patient’s condition crosses the diagnostic threshold (in green), the clinician prescribes the treatment (in yellow) and treatment review (in red) thresholds. The patient self-monitors the parameters of their condition and enters these into the LHS application. If assessment and prediction of their condition rises above the treatment threshold, the patient receives treatment, be it medication or otherwise. If it rises above the treatment review threshold, the clinician is alerted that the patient requires review so that an appointment can be offered.

To fulfil PamBayesian’s aims the following LHS types from the LHS pathway, which apply to the levels of *medical practice* from the application pathway (in brackets), are needed:

1. *Cohort identification—learning evidence* (EBM) and operating within the context of the *learning healthcare organisation* of the *system pathway* to identify patients with similar demographic and clinical characteristics.
2. *Clinical decision support system—specific to patient* (precision medicine) to collect and analyse daily data and operating within the *LHS* level of the *system pathway* to provide relevant patient feedback.
3. *Predictive patient risk modelling—specific to patient* (precision medicine) and operating within the *LHS* level of the *system pathway* to predict and identify potential future adverse events.

### LHS in the context of empowering patient participation in healthcare

Despite advances in modern medicine, many chronic conditions such as diabetes and rheumatoid arthritis have generally proven incurable. The daily life of patients with chronic conditions is highly affected by disease progression; over time disease symptoms exacerbate until they overwhelm the patient. Patients must constantly evaluate their condition, making day-to-day decisions regarding care and relying on advice from their treating clinicians to guide those decisions. Again, despite medical advances, access to healthcare remains a significant issue for all patients. Regular appointments with doctors or nurses are time consuming, expensive, inconvenient and, in many cases, cannot be scheduled to coincide with times when the worst symptomatology may present.

**Figure 7:**
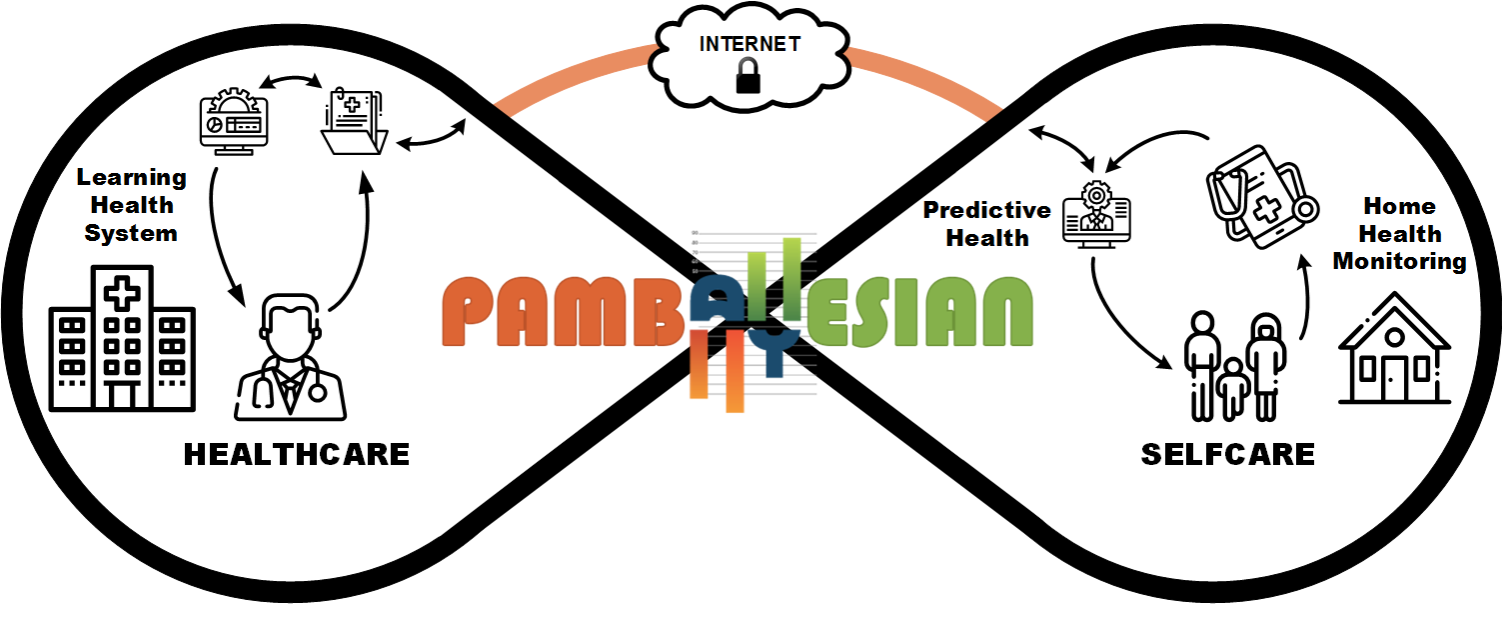
Using PamBayesian to promote patient empowerment.

PamBayesian aims to empower patients to undertake day-to-day self-care within boundaries; diagnostic, treatment and treatment review thresholds that are defined by the patient’s clinician. As shown on the right side of figure 7, home health monitoring devices and applications will be used to gather patient symptoms, measurements and reports about their condition, and with BN intelligence will tailor clinical knowledge and generate patient advices. In this way, PamBayesian promotes continuous monitoring of the patient’s condition while supporting patient self-management and engagement of timely interventions. PamBayesian also promotes a more effective and efficient interaction model between patients and clinicians whereby expensive and time-consuming clinic visits need only occur when a patient’s monitoring shows that their symptomatology has escalated and surpassed the *treatment review* threshold as discussed in the previous section.

To fulfil PamBayesian’s aims the following LHS types from the LHS pathway, which apply to the levels of *medical practice* from the application pathway (in brackets), are needed:

1. *Cohort identification—learning evidence* (EBM) and operating within the context of the *learning healthcare organisation* of the *system pathway* to identify patients with similar demographic and clinical characteristics.
2. *Clinical decision support system—specific to patient* (precision medicine) to collect and analyse daily data and operating within the *LHS* level of the *system pathway* to provide relevant patient feedback.
3. *Predictive patient risk modelling—specific to patient* (precision medicine) and operating within the *LHS* level of the *system pathway* to predict and identify potential future adverse events.

## Conclusion, summary and future work

LHS are a recent concept with little more than a decade of research but limited exposure. LHS have the potential to completely change the way medicine is practised by guiding treatment selection on characteristics of the individual patient (precision medicine) instead of focusing on the disease (EBM). The conceptual approach and taxonomy for LHS and clinical care specifications were used to develop the unifying learning healthcare organisational model and LHS framework. The *LAGOS framework* presented provides: (1) *clinicians and care providers* with a conceptual tool to establish where and how different LHS apply to, and can be integrated with, their clinical practice; and (2) *researchers*, especially health informaticians, with clear and accurate conceptualisation for use in developing novel LHS solutions. The *conceptualisation* of LHS for the LAGOS framework is incorporated into the learning cycle aimed at constant improvement of patient care through iterative review and development of new knowledge from past experience. This approach also demonstrates how under-representation of any one aspect breaks the cycle and leads to the entire health system becoming ineffective. Thus, the LAGOS framework is for unifying four things: (1) health technology, (2) care specifications, (3) the learning health organisation, and (4) LHS. Furthermore, the LAGOS framework identifies where each LHS type applies in clinical practice, and how each pathway focuses care towards the individual patient (*precision medicine*).

Future work that will aid in continued development and expansion of the LHS domain includes development of a meta-model, which will require: (1) investigation to identify a taxonomy of computational approaches used in the domain; and (2) identification of common developmental properties within those LHS that have been either built and tested or implemented. In this way, the domain can begin to comprehend those few LHS that have been implemented and develop strategies to investigate the properties of LHS that are more likely to be of clinical benefit or which can be said to have participated in delivering the sought-after quality, safety, efficiency and efficacy aspects.

## References

[1] Friedman C, Rubin J, Brown J, et al Toward a science of learning systems: a research agenda for the high-functioning learning health system. J Am Med Inform Assoc 2015;22:43–50. 10.1136/amiajnl-2014-002977 25342177 PMC4433378

[2] English M, Irimu G, Agweyu A, et al Building learning health systems to accelerate research and improve outcomes of clinical care in low- and middle-income countries. PLoS Med 2016;13:e1001991 10.1371/journal.pmed.1001991 27070913 PMC4829240

[3] Mullins CD, Wingate La'Marcus T, Edwards HA, et al Transitioning from learning healthcare systems to learning health care communities. J Comp Eff Res 2018;7:603–14. 10.2217/cer-2017-0105 29478331

[4] Nwaru BI, Friedman C, Halamka J, et al Can learning health systems help organisations deliver personalised care? BMC Med 2017;15 10.1186/s12916-017-0935-0 PMC5623976 28965492

[5] McLachlan S, Potts HWW, Dube K, et al The Heimdall framework for supporting characterisation of learning health systems. J Innov Health Inform 2018;25 10.14236/jhi.v25i2.996 30398449

[6] McLachlan S, Dube K, Buchanan D, et al Learning health systems: the research community awareness challenge. J Innov Health Inform 2018;25 10.14236/jhi.v25i1.981 29717954

[7] McLachlan S, Dube K, Johnson O, et al A framework for analysing learning health systems: are we removing the most impactful barriers? Learning Health Systems 2019;25 10.1002/lrh2.10189 PMC6802533 31641685

[8] McLachlan S, Kyrimi E, Dube K, et al Clinical caremap development: How can caremaps standardise care when they are not standardised? Paper presented at the 12th International Joint Conference on Biomedical Systems and Technologies (BIOSTEC 2019), Czech Republic, 2019.

[9] McLachlan S, Kyrimi E, Dube K, et al A taxonomy to assist standardisation of evidence-based clinical care process specification documents. manuscript submitted to the SAGE health informatics Journal. research report. London, UK: Queen Mary University of London, 2019.

[10] PamBayesian The PamBayesian project, 2018 Available: http://www.pambayesian.org

[11] Beckmann JS, Lew D Reconciling evidence-based medicine and precision medicine in the era of big data: challenges and opportunities. Genome Med 2016;8 10.1186/s13073-016-0388-7 PMC5165712 27993174

[12] Joffe H, Yardley L Content and thematic analysis. research methods for clinical and health psychology. 56, 2004.

[13] Vaismoradi M, Jones J, Turunen H, et al Theme development in qualitative content analysis and thematic analysis. J Nurs Educ Pract 2016;6:100–10. 10.5430/jnep.v6n5p100

[14] Fereday J, Muir-Cochrane E Demonstrating rigor using thematic analysis: a hybrid approach of inductive and deductive coding and theme development. Int J Qual Methods 2006;5:80–92. 10.1177/160940690600500107

[15] Vaismoradi M, Turunen H, Bondas T Content analysis and thematic analysis: implications for conducting a qualitative descriptive study. Nurs Health Sci 2013;15:398–405. 10.1111/nhs.12048 23480423

[16] Sperling D Assessment of technological choices using a pathway methodology. Transportation Research 1984;18:343–53. 10.1016/0191-2607(84)90172-9

[17] Horwitz RI, Hayes-Conroy A, Caricchio R, et al From evidence based medicine to medicine based evidence. Am J Med 2017;130:1246–50. 10.1016/j.amjmed.2017.06.012 28711551

[18] Masic I, Miokovic M, Muhamedagic B Evidence based medicine - new approaches and challenges. Acta Inform Med 2008;16 10.5455/aim.2008.16.219-225 PMC3789163 24109156

[19] Cable G Integrating case study and survey research methods: an example in information systems. Eur J Inform Syst 1994;3.

[20] Lee AS A scientific methodology for MIS case studies. MIS Quarterly 1989;13:33–50. 10.2307/248698

[21] Trellis W Application of a case study methodology. The Qualitative Report 1987;3.

[22] Yin R Applications of case study research. Sage, 2011.

[23] Baxter P, Jack S Qualitative case study methodology: study design and implementation for novice researchers. The Qualitative Report 2008;13.

[24] Benbasat I, Goldstein DK, Mead M The case research strategy in studies of information systems. MIS Quarterly 1987;11:369–86. 10.2307/248684

[25] IoM The learning healthcare system: workshop summary, 2007 https://www.ncbi.nlm.nih.gov/pubmed/21452449 21452449

[26] IoM Digital infrastructure for the learning health system: the foundation for continuous improvement in health and health care. Washington DC, 2011 https://www.ncbi.nlm.nih.gov/pubmed/22379651 22379651

[27] UNC Medical residents: EBM review and practice, 2017 Available: https://guides.lib.unc.edu/residents/review-evidence

[28] Friedman C, Allee N, Delaney B, et al The science of learning health systems: foundations for a new Journal. learning health systems, 2016. 10.1002/lrh2.10020 PMC6516721 31245555

[29] Krumholz HM Big data and new knowledge in medicine: the thinking, training, and tools needed for a learning health system. Health Aff 2014;33:1163–70. 10.1377/hlthaff.2014.0053 PMC5459394 25006142

[30] Faden R, Kass N, Goodman S, et al An ethics framework for a learning health care system: a departure from traditional research ethics and clinical ethics, 2013. 10.1002/hast.134 23315888

[31] Harper E Can big data transform electronic health records into learning health systems? Nursing Informatics 2014;201:470–5. 24943583

[32] Forrest CB, Margolis P, Seid M, et al PEDSnet: how a prototype pediatric learning health system is being expanded into a national network. Health Aff 2014;33:1171–7. 10.1377/hlthaff.2014.0127 25006143

[33] Friedman CP, Wong AK, Blumenthal D Achieving a nationwide learning health system. Sci Transl Med 2010;2:1–3. 10.1126/scitranslmed.3001456 21068440

[34] Lavigne JE, Brown J, Matzke GR Population health and medicine: policy and financial drivers. Am J Health Syst Pharm 2017;74:1413–21. 10.2146/ajhp161051 28887343

[35] Agrawal P, Kosowsky JM Clinical practice guidelines in the emergency department. Emerg Med Clin North Am 2009;27:555–67. 10.1016/j.emc.2009.07.001 19932391

[36] Koon AD, Hawkins B, Mayhew SH Framing and the health policy process: a scoping review. Health Policy Plan 2016;31:801–16. 10.1093/heapol/czv128 26873903 PMC4916318

[37] Gray M, Ricciardi W From public health to population medicine: the contribution of public health to health care services. Eur J Public Health 2010;20:366–7. 10.1093/eurpub/ckq091 20660174

